# Unraveling the impact of human cerebrospinal fluid on human neural stem cell fate

**DOI:** 10.3389/fcell.2025.1527557

**Published:** 2025-03-13

**Authors:** Klaudia Radoszkiewicz, Aleksandra Bzinkowska, Monika Sypecka, Dorota Sulejczak, Daniela Ferrari, Maurizio Gelati, Angelo Luigi Vescovi, Anna Sarnowska

**Affiliations:** ^1^ Translational Platform for Regenerative Medicine, Mossakowski Medical Research Institute, Polish Academy of Sciences, Warsaw, Poland; ^2^ Department of Experimental Pharmacology, Mossakowski Medical Research Institute, Polish Academy of Sciences, Warsaw, Poland; ^3^ Department of Biotechnology and Biosciences, University of Milano-Bicocca, Milan, Italy; ^4^ UPTA Unit Fondazione IRCCS Casa Sollievo della Sofferenza, Foggia, Italy; ^5^ IRCCS Casa Sollievo della Sofferenza, Foggia, Italy

**Keywords:** neural stem cells, ischemic stroke, cerebrospinal fluid, neuroprotection, cell therapy

## Abstract

Human neural stem/progenitor cells (hNSCs) can potentially treat neurological diseases, but their low survival and proliferation rates after transplantation remain challenging. In our study, we preincubated hNSCs with the human cerebrospinal fluid (CSF) to obtain closer to the physiological brain environment and to assess NSC fate and their therapeutic abilities *in vitro*, *ex vivo*, and *in vivo*. We observed significant changes in the differentiation, migratory, and secretory potential of CSF-treated hNSCs, as well as their elevated neuroprotective potential after co-culture with ischemically damaged by oxygen-glucose deprivation (OGD) organotypic rat hippocampal slices culture (OHC) in comparison to the cells cultured in the standard conditions. Next, we investigated their survival and anti-inflammatory abilities in an *in vivo* ouabain-induced stroke model. This study highlighted and confirmed the critical importance of nutritional supplementation in maintaining NSC culture and enhancing its therapeutic properties.

## 1 Introduction

After several decades, we still have not obtained sufficient clinical results that could significantly improve the lives of neurological patients (Grochowski et al., 2018). One of the most commonly occurring neurological diseases that has taken much interest is ischemic stroke. Its consequences often dramatically lower the life quality of patients, resulting in disability or even leading to death. Much hope is given to endogenous neural stem cells, which can, due to the activation, migrate and subsequently participate in restoring the damaged brain area functions after stroke. However, due to their relatively limited amount and survival, the recovery is still insufficient and the injury-induced neurogenesis is not successful (Kokaia and Lindvall, 2003; Liu et al., 2009; Kernie and Parent, 2010; Kokaia and Darsalia, 2018). Thus, using exogenous NSCs holds promise for the repair of ischemically damaged neural tissue (Park et al., 2002; Andres et al., 2008; Steinberg et al., 2016; Boese et al., 2018). In this case, exogenous NSCs, among other cell types, can further selectively differentiate into all neural lineages and present minimal tumorigenic risk (Rakic, 2009; Yan et al., 2013; Beattie and Hippenmeyer, 2017). Moreover, they were already seen to be very supportive serving as chaperone cells for the injured/dysfunctional tissue (Hess and Borlongan, 2007). Despite all aforementioned properties suggesting a successful use in treating many diseases, achieving such a goal in clinical trials seems to be still elusive. To a great degree, this can be caused by their limited ability to regenerate. Low cell survival and proliferation rate after transplantation are one of the biggest issues (Lee et al., 2017; Hayashi et al., 2020). The mechanisms that occur after the cell injection are still not well-defined (Boese et al., 2018; Ottoboni et al., 2020). Along with still unsatisfying knowledge regarding numerous processes appearing in the accompaniment of specific factors of the brain niche, even at the preclinical level, the analysis of the results remains very difficult (Gil-Perotín et al., 2013). Reflecting the exact physicochemical and spatial conditions of the neural niche *in vitro* is currently debatable. We pointed out previously that the wide variation of medium composition used by each research group limits reliable comparison of the results and their interpretation (Radoszkiewicz et al., 2023c; 2023b). As a consequence, the mechanisms that are involved in *in vivo* cell application still need to be widely explored.

**FIGURE 1:**
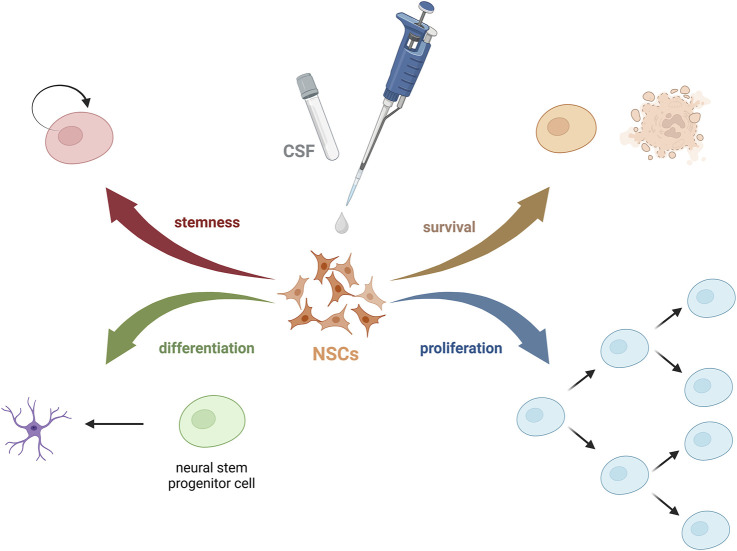
Possible effects of CSF on NSCs.

The behavior of NSCs is highly dependent on the surrounding microenvironment. The niche regulates its fate *via* biochemical *stimuli* (such as growth factors, hormones, or peptides), biophysical factors (pressure, shear stress, geometry), and cell-cell interactions. These components have a great influence on the adhesion, survival, proliferation, migration, morphology, and differentiation of stem cells. Through our exploration of factors influencing the poor fate of exogenous cells post-transplantation, especially after the intrathecal injection method, we turned our focus to the cerebrospinal fluid (CSF). While CSF has traditionally been seen as a fluid with basic physiological and mechanical functions, recent studies underlie its critical role in complex brain physiology, particularly during development, influencing the functions of NSCs and brain restoration (Wichmann et al., 2022). Despite its importance as a diagnostic tool, the components of CSF and their specific roles remain under-explored (Figure 1). What is more, after analyzing so far published papers on the effect of CSF on stem cells, we have seen that it varies between performed research, what hampers the ability to compare them, and coming to uniform conclusions (Radoszkiewicz et al., 2023a). Nonetheless, limited research on the effects and involved mechanisms of unaltered or naturally altered CSF on neurogenesis emphasizes the need for further studies. This, however, allows exploring still not well-known areas of human brain function and offers a glimpse of hope of creating a laboratory environment that closely mimics the physiological brain niche.

## 2 Materials and methods

### 2.1 Neural stem cell culture

Human fetal neural stem/progenitor cells (hNSCs) were obtained from the IRCCS Casa Sollievo della Sofferenza, Viale Cappuccini 1, San Giovanni Rotondo, 71,013 Foggia, Italy. The material was acquired in full compliance with legal and ethical standards, adhering to current local informed consent procedures. The cells were isolated from the fetal human brain following previously described protocols (Vescovi et al., 2009; Gelati et al., 2013). HNSCs were cultured in standard culture conditions in humidified incubators under 5% O_2_ and 5% CO_2_ conditions, at 37°C. The medium containing DMEM/F12 (Gibco, Thermo Fisher Scientific, Waltham, MA, USA), GlutaMAX® (1%, Gibco), Penicillin/streptomycin (1%, Gibco), Heparin (0.1%, Sigma-Aldrich, Saint Louis, MO, USA), N2 supplement® (1%, Gibco), B27 supplement® (2%, Gibco), EGF (20 ng/mL, Gibco), bFGF (20 ng/mL, Gibco) was replaced every 3 days. The neurospheres (3D culture) were mechanically dissociated when they gained the desired diameter. The cells in 2D culture were seeded into 24-well or 96-well plates covered with Poly-d-lysine and laminin and the standard culture medium without growth factors was used. Over the past years, the team members have comprehensively characterized used in the manuscript NSCs line regarding the markers, differentiation potential, and biological activity (Mazzini et al., 2015; Rosati et al., 2018; Profico et al., 2022).

#### 2.1.1 Cerebrospinal fluid (CSF)

Human CSF samples were collected from 20 adult volunteers following routine diagnostic procedures conducted at the Department of Neurology, Medical University of Warsaw, Poland. The collection process adhered to the ethical guidelines outlined by the Medical University of Warsaw ethics committee (guideline AKBE/242/2022). These CSF samples were classified as leftovers, as they were no longer required for further medical diagnostics (neurological diseases were ruled out based on the CSF analysis). The leftover CSF samples were pooled, and selected analytes were analyzed using a Luminex assay. To assess the impact of CSF on NSCs, cells were cultured in a medium supplemented with 30% CSF, which was the maximal volume necessary for the planned experiments and their repetitions. For proliferation and migration assessments, cells were also cultured in 100% CSF. NSCs were cultured in the CSF-containing medium for up to 7 days.

#### 2.1.2 NSC scratch injury

To evaluate the influence of CSF on NSC migration, a scratch assay was performed using culture inserts (ibidi). The experiment was conducted in triplicate, with 10,000 cells seeded into each chamber of the inserts. After 24 h, the inserts were removed, and each well was filled with one of the following media: standard culture medium without growth factors (control), medium supplemented with 30% CSF (30% CSF), or 100% CSF (100% CSF). Images of the scratch area were captured at 0, 2, 24, and 48 h after performing the scratch. At the end of the experiment, the cells were fixed with 4% paraformaldehyde (PFA; Sigma) and stained using a neurite outgrowth assay kit to visualize neurites. Three random pictures of each scratch injury were captured from each well of the 24-well plate using Cell Observer (Carl Zeiss, Oberkochen, Germany) inverted microscope and ZEN software (Carl Zeiss, Oberkochen, Germany). At least four wells were prepared for each variant.

#### 2.1.3 Neurite outgrowth assay

To visualize the neurites in the scratch area, the cells were stained using a Neurite Outgrowth Kit (Thermo Fischer Scientific). Next, the neurites were analyzed using Fiji (formerly ImageJ) software. A region of interest (ROI) was marked around the scratch, excluding the edges and neuronal bodies, while the integrated density was used to calculate a sum of all pixels within the ROI. To obtain the percentage of ROI covered with neurites we divide the integrated density/area of ROI. These experiments were carried out using a 24-well plate. At least four wells were prepared for each variant.

#### 2.1.4 Cell proliferation assay

The cell proliferation rate was assessed using the PrestoBlue™ Cell Viability Reagent (Invitrogen, Thermo Fisher, Rochester, NY, USA) following the manufacturer’s protocol. The cells were cultured in a standard culture medium without growth factors. HNSCs were incubated with this reagent for 1 h in the dark, on Day 1, Day 3, and Day 7 of monolayer culture performed on a 96-well poly-L-lysine/laminin-coated plate. The cell density was 10,000 cells/well. For analysis, the starting point (Day 1) was set as the baseline at 100%. Absorbance was measured with the use of a microplate reader, Omega PlateReader (BMG LABTECH), at a 590–600 nm wavelength.

#### 2.1.5 Organotypic rat hippocampal slice culture (OHC)

For *ex vivo* experiments, 7-day-old Wistar rats (12 animals) were obtained from the Mossakowski Medical Research Institute Breeding House. All experiments were approved by the Ethical Committee and conducted in accordance with ethical guidelines. The experiment was conducted following the Stoppini method that was previously modified in our lab (Stoppini et al., 1991; Figiel-Dabrowska et al., 2021). All procedures were performed on ice. In brief, following decapitation, the rat hippocampi were isolated and sectioned into 400 µm slices using a McIlwain tissue chopper (Ted Pella, Poznan, Poland) into 400 µm slices. The selected slices were transferred onto the organotypic culture membranes (Millipore, Concord Road Billerica, MA, USA), placed on 6-well plates (ThermoFischer Scientific) and filled with 960 µL of the medium consisting of DMEM/F12 (Gibco), 25% HBSS (Gibco), HEPES (Gibco), 5 mg/mL glucose (Sigma-Aldrich) and 1% of antibiotic-antimycotic solution (Gibco). The rat organotypic hippocampal slice cultures (OHC) were maintained for 7 days at 34°C, in an atmosphere of 5% O_2_ and 5% CO_2_. After the incubation period, the OHCs were utilized for co-culture experiments with various NSC culture conditions.

#### 2.1.6 Oxygen-glucose deprivation (OGD)

**FIGURE 2:**
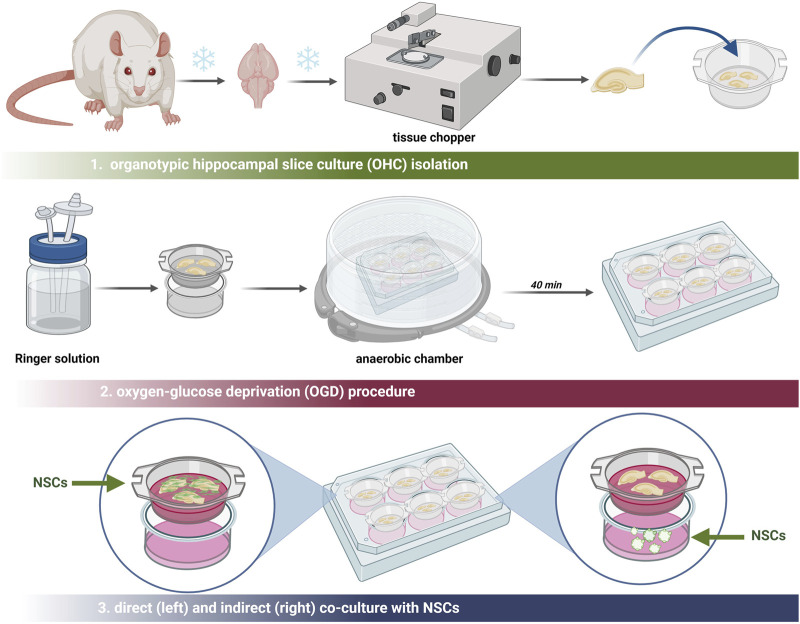
Schematic overview of all procedures executed in the *ex vivo* model.

The procedure of oxygen-glucose deprivation (OGD) was described previously (Figiel-Dabrowska et al., 2021) (Figure 2). After 7 days of organotypic hippocampal culture (OHC), preselection of the hippocampal slices was carried out using propidium iodide (PI; Thermo Fisher Scientific) to identify slices with damage in the CA1 region. Damaged slices were discarded. The culture medium was then replaced with deoxygenated Ringer’s solution (Sigma-Aldrich) supplemented with mannitol (Sigma-Aldrich). The membranes with the selected slices were transferred into a hypoxic chamber for oxygen deprivation for 40 min at 34°C, 5% O_2,_ and 5% CO_2_ conditions. Subsequently, the membranes were washed in PBS (ThermoFisher Scientific) and the co-culture with all cell variants was conducted. For indirect co-culture, the neurospheres were seeded into the 6-well plate, under the membrane with the slices. To perform the direct co-culture, the cells in the suspension were directly seeded onto the slice. To analyze the neuroprotective effect of hNSCs, 24 h after this procedure, the slices were stained with PI (ThermoFisher Scientific) for 30 min and CA1-region hippocampal cell death was investigated. The images of the CA1 region were taken using an LSM 510 confocal microscope (Zeiss).

#### 2.1.7 Ouabain-induced brain injury and hNSC transplantation

**FIGURE 3:**
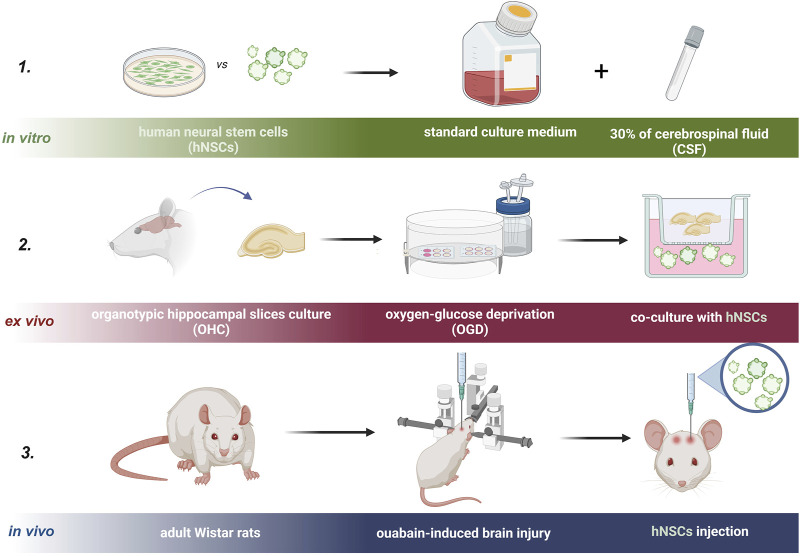
Experimental design of the study is presented in three steps. Step one: *in vitro*, step two: *ex vivo*, and step three: *in vivo* experiment.

For *in vivo* experiments, 3-month-old male 250g-weight Wistar rats from the Mossakowski Medical Research Institute Animal Breeding House were used (Figure 3). Approval for this study was granted by the Local Ethical Committee in Warsaw, Poland (No. WAW2/147/2022). For each experiment, six animals were used (n = 6).

To induce focal brain damage, the rats underwent ouabain-induced brain injury. The procedure involved anesthetizing the rats with ketamine (10%; 90 mg/kg body weight, administered intraperitoneally) and xylazine (2%; 10 mg/kg body weight, administered intraperitoneally). Subsequently, the rats were positioned in a stereotactic apparatus (Stoelting). A small hole was drilled in the cranium on the right hemisphere at the following coordinates (A 0.0, L 3.0, D 5.0 mm). Using a micro infusion pump (Stoelting) and a 10-μL Hamilton syringe (Hamilton) equipped with a 15-mm-long needle (gauge 33, Hamilton), 1 μL of 5 mmol ouabain (Sigma) was injected into the brain at a rate of 0.1 μL/min. To minimize brain shift, a 5-min delay was introduced between the needle insertion and the actual injection. Subsequently, the needle was carefully removed, and the incision in the skin was closed using sutures. Post-procedure, the rats received antibiotics and painkillers (Baytril and Metacam, 5 mg/kg, administered intraperitoneally) and were housed appropriately for recovery.

Two days following the ouabain-induced brain injury, the rats underwent a second anesthesia for cell transplantation. The stereotaxic surgery was conducted under the influence of ketamine (10%; 90 mg/kg body weight, administered intraperitoneally) and xylazine (2%; 10 mg/kg body weight, administered intraperitoneally). NSCs were transplanted using a 25-μL Hamilton syringe into the corpus callosum. The injection was controlled by a micro infusion pump at a rate of 0.5 μL/min. The coordinates employed were A 0.0, L 4.0, and V 3.0, with the bregma adjusted to the same horizontal plane, and ventral coordinates calculated from the dura. Upon removal of the needle, the skin was securely resealed with sutures (Braun). Subsequently, the rats received antibiotics and painkillers (Baytril and Metacam, 5 mg/kg body weight, administered intraperitoneally) and were housed.

Seven days post-transplantation, the animals were euthanized for immunohistochemical analysis. The rats were anesthetized and subsequently decapitated. Following decapitation, the brains were isolated and fixed in 4% paraformaldehyde (PFA; Sigma). The fixed brains were then suspended in 30% sucrose for cryoprotection and stored at −80°C for cryopreservation. The day prior to cryosectioning, the brains were transferred to −20°C and subsequently sectioned into 20 µm slices using a cryostat. The sections were stored at −80°C until immunostaining procedures were performed. This approach facilitated the examination of the transplanted cell locations and their interactions with the damaged neural tissue.

### 2.2 Results analysis

#### 2.2.1 Immunofluorescence (IF) staining

For IF staining, we used the previously described technique for 2D and 3D culture (Radoszkiewicz et al., 2023c). In 2D, after experiments, the cells were deprived of culture medium, washed twice in PBS (ThermoFisher Scientific), and fixed with 4% PFA (ThermoFisher Scientific) for 15 min, at room temperature (RT). Subsequently, the cells were permeabilized in 0.2% Triton X-100 (Sigma-Aldrich) for 30 min. To prevent nonspecific bindings, a mixture of 10% Goat Serum (GS, Gibco) in a 1% Bovine Serum Albumin (BSA, Sigma-Aldrich) solution was applied for 1 h at RT. Following this, the cells were washed again with PBS (ThermoFisher Scientific) and incubated with primary antibodies (Table 1) overnight at 4°C. Next, the cells were washed in PBS (ThermoFisher Scientific) and incubated with secondary antibodies (Table 2) in the dark for 1 h, at RT. The next step involved mounting the coverslips with Fluoromount-G with DAPI (ThermoFisher Scientific). The staining protocol for neurospheres and OHC slices was similar, but a blocking mixture was prepared using 6% BSA solution (Sigma-Aldrich), 0.1% Triton X-100 (Sigma-Aldrich) in PBS (Sigma-Aldrich). Moreover, the neurospheres were stained with 1% Hoechst 33,342 (Sigma-Aldrich) solution for 30 min to visualize the cell nuclei. The stained neurospheres were transferred to the surface of the microscope slide and a single drop of the mounting medium was applied to then close it with a 14 mm-diameter coverslip. The slides were left at RT for overnight drying. To assess cell survival, migration, differentiation, and local immune response *in vivo*, immunostaining procedures, as outlined above were performed. The presence of specific markers, such as Ki67 for proliferation, and Nestin, GFAP for neural differentiation, was determined. The host immune response was analyzed using goat polyclonal anti-ionized calcium-binding adapter molecule 1 (Iba1) antibody for microglia. Additionally, the presence of transplanted NSCs in rat brain tissue was investigated using anti-nuclear mitotic apparatus protein (anti-mitochondria antibody-a-mit). The brain sections were permeabilized and blocked using 6% BSA solution (Sigma-Aldrich), 0.1% Triton X-100 (Sigma-Aldrich) in PBS (Sigma-Aldrich) for 90 min at RT. After washing in PBS, the slices were incubated with primary antibodies overnight at 4°C. Then, they were washed in PBS again and secondary antibodies were incubated for 1 h. 1% of Hoechst 33,342 (Sigma-Aldrich) solution was used to visualize the cell nuclei. The slices were closed with the mounting medium (Dako).

**TABLE 1:** Primary antibodies used for IF staining.

Antigen	Source	Isotype	Dilution	Manufacturer	Catalog number
GFAP	Rabbit polyclonal	IgG	1:400	Dako	Z0334
Ki-67	Rabbit polyclonal	IgG	1:200	Abcam	AB15580
β-Tubulin III	Mouse monoclonal	IgG2b	1:1,000	Sigma-Aldrich	T8660
A2B5	Mouse monoclonal	IgM	1:200	Millipore	MAB312R
Nestin	Mouse monoclonal	IgG1	1:500	Millipore	MAB5326
NG2	Rabbit polyclonal	IgG	1:150	Millipore	AB5320
SOX2	Rabbit polyclonal	IgG	1:150	Sigma-Aldrich	SAB5700644
S100*β*	Rabbit polyclonal	IgG	1:100	Abcam	AB52642
a-mit	Mouse monoclonal	IgG1	1:100	Abcam	AB92824
MAP2	Mouse monoclonal	IgG1	1:1,000	Sigma-Aldrich	N4403
Iba-1	Goat Polyclonal	IgG	1:400	Abcam	AB5076

**TABLE 2:** Secondary antibodies used for IF staining. The presented dilutions were half-reduced for immunohistochemical stainings.

Antigen	Fluorochrome	Isotype	Dilution	Manufacturer	Catalog number
Alexa Fluor Goat (anti-rabbit)	Alexa 488	IgG	1:1,000	Life Technologies	A11034
Alexa Fluor Goat (anti-rabbit)	Alexa 546	IgG	1:1,000	Life Technologies	A11035
Alexa Fluor Goat (anti-mouse)	Alexa 546	IgM	1:1,000	Life Technologies	A21123
Alexa Fluor Goat (anti-mouse)	Alexa 546	IgG2b	1:1,000	Life Technologies	A21143
ALEXA FLUOR GOAT (ANTI-RABBIT)	Alexa 647	IgG	1:1,000	Life Technologies	A21245
ALEXA FLUOR (ANTI-GOAT)	Alexa 546	IgG	1:1,000	Life Technologies	A11056

For each variant, a negative control to eliminate non-specific reactions was executed. The results were analyzed using an LSM 780 confocal microscope (Carl Zeiss). For quantitative analysis, the calculation of positively stained 2D culture cells relative to the entire population was conducted using ZEN 2 Blue Edition software (Carl Zeiss). At least three repetitions were made for each experimental variant.

#### 2.2.2 CMFDA (5-Chloromethylfluorescein diacetate) staining

NSC migration on the hippocampal slices *ex vivo* was visualized by a previous 30-min incubation at 37 °C with 10 mM of green CMFDA (abcam). Next, NSCs suspended in PBS were transferred onto the slices and analyzed after 7, 14, and 21 days using fluorescent microscopy with Zeiss Axiovert A.1 fluorescent microscope (Carl Zeiss) and LSM 780 confocal laser scanning system and ZEN software (Carl Zeiss).

#### 2.2.3 qRT-PCR analysis

The gene expression analysis was performed with the use of qRT-PCR analysis. Total RNA was isolated from the cells using fenozol, according to the protocol instructions (A&A Biotechnology, Gdansk, Poland). The purity of each sample and RNA concentration was measured by NanoDrop ND-1000 (Thermo Scientific, Thermo Fischer Scientific). Then, the cDNA was obtained by reverse transcription conducted with a High-Capacity RNA-to-cDNA Kit (Applied Biosystems, Thermo Fischer Scientific). cDNA probes were diluted in RNase-free H_2_O (Sigma-Aldrich) and stored at −20°C until further use. Quantitative RT-PCR reaction was performed on Fast 7,500 Thermocycler (Applied Biosystems) with 10 ng of cDNA in 15 µL with the use of 3-color RT HS-PCR SYBR Green Master Mix (Applied Biosystems) and specific primers for *KI67* (proliferation) *MAP2, GFAP, NESTIN, NG2,* and *β-TUBULIN-III (*neural differentiation) with *RPLP0* as a reference gene (Table 3). The final relative gene expression was calculated by the 2^−ΔΔCt^ method which was described previously (Figiel-Dabrowska et al., 2021; Rybkowska et al., 2023).

**TABLE 3:** Primer sequences used with qRT-PCR.

Gene	NCBI reference Sequence	Product size	Primer sequence (5’ -> 3′)
*RPLP0*	NM_001002.4	1,229 bp	F: 5′AAAATCTCCAGGGGCACCATT3′ R: 5′GCTCCCACTTTGTCTCCAGT3′
*KI67*	NM_002417	12,507 bp	F: GAAAGAGTGGCAACCTGCCTTC R:GCACCAAGTTTTACTACATCTGCC
*MAP2*	NM_001375545.1	99 bp	F: TTGGTGCCGAGTGAGAAGA R: GTCTGGCAGTGGTTGGTTAA
*GFAP*	NM_001363846.2	100 bp	F: CCGACAGCAGGTCCATGT R: GTTGCTGGACGCCATTG
*NESTIN*	NM_006617.2	64 bp	F: GGGAAGAGGTGATGGAACCA R: AAGCCCTGAACCCTCTTTGC
*NG2*	NM_001897.5	118 bp	F: GTCTACACCATCGAGCAGCC R: TGTGTGAGAACAGCACGAGC
*β-TUBULIN-III*	NM_001197181.2	126 bp	F: GGAAGAGGGCGAGATGTACG R: GGGTTTAGACACTGCTGGCT

#### 2.2.4 Luminex cytokine and chemokine assay

To investigate the concentration of the selected analyte in the medium samples, Twelve-plex Human Magnetic Luminex Assays (R&D Systems, cat. no. LXSAHM-12) were used. We analyzed the following variants of the culture medium, at selected time points (24 h, 7 days, or 14 days of experiment).

-control–NSCs cultured in the standard medium
-CSF- NSCs cultured in standard medium with 30% of CSF addition
-NSC OGD–NSCs in standard medium co-cultured with OHC after OGD
-NSC CSF OGD- NSCs cultured in standard medium with 30% of CSF addition co-cultured with OHC after OGD

In addition, the media without cells were analyzed. The concentration of Angiogenin, BDNF, CCL2/JE/MCP-1, FGF basic/FGF2/bFGF, EGF, HGF, VEGF, VEGF-C, HGF, ICAM-1, LIF and beta-NGF was investigated using Luminex-based platform and Luminex 200 IS V2.1 Software (Bio-Rad, Hercules, CA, USA). Standard curves were obtained from the reference cytokine gradient concentrations. All the samples were stored at −80 °C before the analysis. All procedures were made on ice. Each sample was frozen/thawed only once.

### 2.3 Statistical analysis

The data are presented as the mean ± SD for n ≥ 3. One-way analysis of variance (ANOVA test) was used to conduct multi-group comparisons, followed by the Tukey test as *post hoc* statistical analysis for each group. The values were considered significant with *p* < 0.05. All statistical analyses of the raw data were performed using GraphPad Prism9.

## 3 Results

### 3.1 Analysis of neurogenic and secretory potential of hNSCs in response to the presence of CSF

**FIGURE 4:**
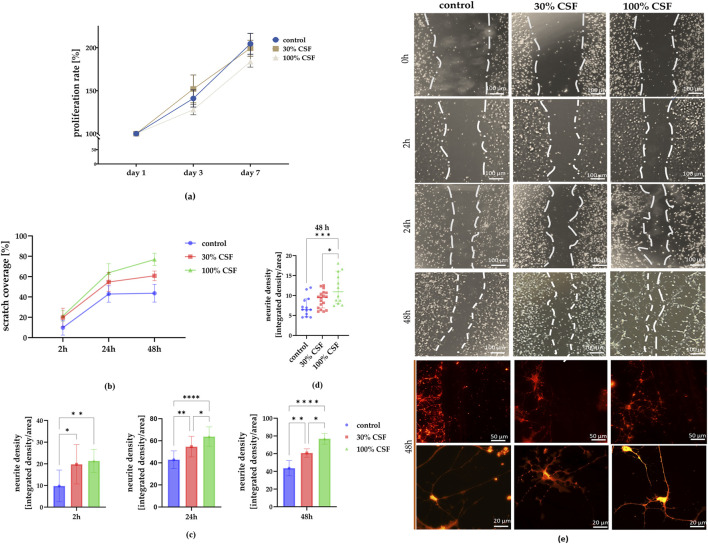
**(a)** The impact of CSF on hNSC proliferation rate (n = 5) after days 1, 3, and seven of culture. **(b)** Determination of neurite outgrowth from scratch injury after 0, 2, 24, and 48 h. Scratch coverage for all culture variants after 2, 24, and 48 h from the injury. **(c)** Changes in scratch coverage between the variants. **(d)** Neurite density after 48 h in all experimental variants. n = 12-18 **(e)** Representative images of NSCs cultured in standard medium (control), medium with 30% addition of CSF, and 100% of CSF. After 48h, the cells were stained using a Neurite Outgrowth Kit. The results of all presented experiments are expressed as mean values ±SD. The differences were considered statistically significant when p-value <0.05. Statistical significance level * for 0.01 < p < 0.05; ** for 0.001 < p < 0.01; **** for p < 0.0001; *** for 0.0001 < p < 0.001; **** for p < 0.0001.

To evaluate the proliferation rate of hNSCs, cells were cultured under standard 2D conditions (medium without growth factors, serving as the control) and treated with either 30% cerebrospinal fluid (30% CSF) or 100% CSF throughout the experiment (Figure 4a). The proliferation analysis revealed a decrease in the 100% CSF group after 7 days (183.8% ± 6.4%), whereas the proliferation rates for the control and 30% CSF groups remained similar (30% CSF: 199.4% ± 9.3%; control: 204.6% ± 12.1%). Thus, the CSF treatment can decrease the proliferation rate of hNSCs.

Next, we investigated whether CSF influences the migratory function of NSCs. Following the removal of the insert-made barrier, cell migration into the space was measured over 48 h (Figure 4e). The control group consistently showed the lowest scratch coverage at each time point (2 h: 9.8% ± 7.3%; 24 h: 42.9% ± 8%; 48 h: 43.6% ± 8.7%) (Figure 4b). Starting from the 2-h time point, the 100% CSF group demonstrated the highest coverage (21.3% ± 5.3%) compared to the 30% CSF group (19.8% ± 9.1%) and the control. This trend became more pronounced after 24 h (100% CSF: 63.8% ± 8.8%; 30% CSF: 54.7% ± 9.3%). Similarly, after 48 h, scratch coverage was approximately 60.8% ± 4.7% for the 30% CSF group and 76.8% ± 6.1% for the 100% CSF group. Additionally, we examined neurite outgrowth in the scratch area at this time point, finding the highest neurite density in the 100% CSF group (100% CSF: 11.77 ± 3.85; 30% CSF: 9.1 ± 2.2; control: 7.23 ± 2.48) (Figures 4c, d). In summary, it seems that CSF can significantly enhance the migratory function and neurite outgrowth of neural stem cells, with the 100% CSF group showing the most visible effects. However, it’s worth noting that the cells treated with 100% CSF started to strongly adhere to each other, prolonging their shape and detaching from the surface. Taken together with the worsened proliferation rate, consequently, the 100% CSF variant was eliminated and both the control and 30% CSF group (hereafter referred to as CSF) were further analyzed for neural and proliferation markers presence, migratory activity, and differences in their secretory profiles.

**FIGURE 5:**
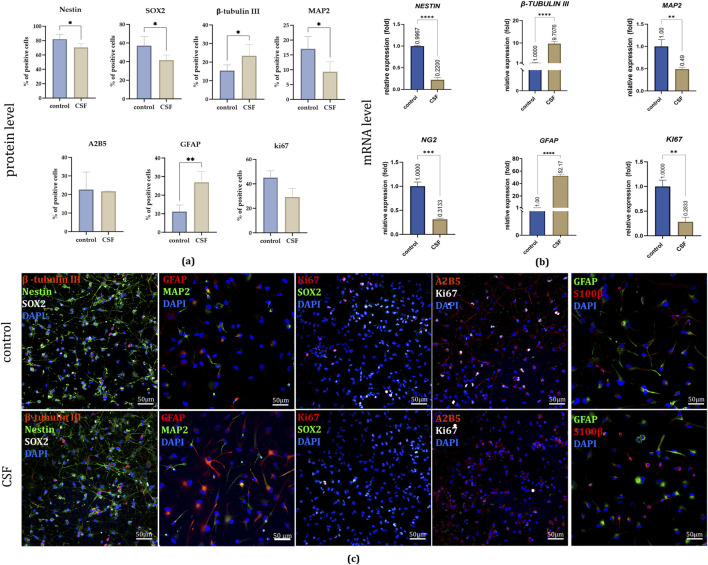
**(a)** The effect of CSF on NSC differentiation and proliferation on day 7. Quantitative analysis of neural lineages and proliferation markers presence. n = 5 **(b)** Relative expression of neural and proliferation genes with RPLP0 as a reference gene and NSC cultured in a standard medium without growth factors as the control group, considered as 1, on day 7. **(c)** Representative images of neural lineages and proliferation markers presence in all experimental variants. The results are presented as mean values of three experiments ±SD. Split-channel images are available in the Supplementary Files. * for 0.01 < p < 0.05; ** for 0.001 < p < 0.01; *** for 0.0001 < p < 0.001; **** for p < 0.0001.

On the protein level, in 2D culture, after 7 days, immunofluorescence staining revealed significant changes in marker expression (Figures 5a, c). The early neural marker NESTIN showed a significant decrease in the CSF group (70.6% ± 5%) compared to the control group (82.1% ± 6.6%). Similarily, we noticed a decrease in SOX2, neural stem and progenitor marker presence for the CSF culture (CSF: 41,5 ± 5.5%, control: 57% ± 10%). Conversely, the early neuronal marker β-TUBULIN III increased markedly in the CSF group (23.4% ± 6.1%) relative to the control (15.4% ± 3.1%). However, the neuronal marker MAP2 was significantly lower in the CSF group (9.3% ± 3.4%) compared to the control (17% ± 4.2%). Furthermore, NSC treatment with CSF resulted in a significant upregulation of GFAP, an astrocytic marker (CSF: 6.9% ± 5.8%; control: 11.2% ± 3.6%). No significant changes were observed in the presence of A2B5+ cells (a marker of NSCs able to differentiate into glia) or KI67+ cells (a marker of proliferation) between the treatment variants. CSF treatment in 2D culture significantly alters the expression of various neural markers, indicating further cell differentiation.

On the mRNA level, the qPCR evaluation of relative gene expression demonstrated results consistent with the observed protein level differences (Figure 5b). Compared to the control group, CSF treatment led to a significant increase in the expression of *GFAP* (52.17 ± 2.54 fold) and *β-TUBULIN III* (9.71 ± 0.93 fold) in hNSCs. Conversely, there was a notable decrease in the expression of *NESTIN* (0.22 ± 0.06 fold) and *MAP2* (0.49 ± 0.04 fold). Additionally, CSF treatment resulted in the downregulation of *NG2*, an oligodendrocyte marker (0.31 ± 0.02 fold), and a similar reduction was observed in *KI67* expression (0.28 ± 0.09 fold) compared to the control. The observation in the CSF group regarding the decreased proliferative potential based on *KI67* expression was also confirmed by their lowered proliferation rate. Thus, CSF significantly modifies the gene expression of hNSCs, enhancing astrocytic and early neuronal differentiation while reducing the expression of markers specific for oligodendrocytes, and proliferation.

**FIGURE 6:**
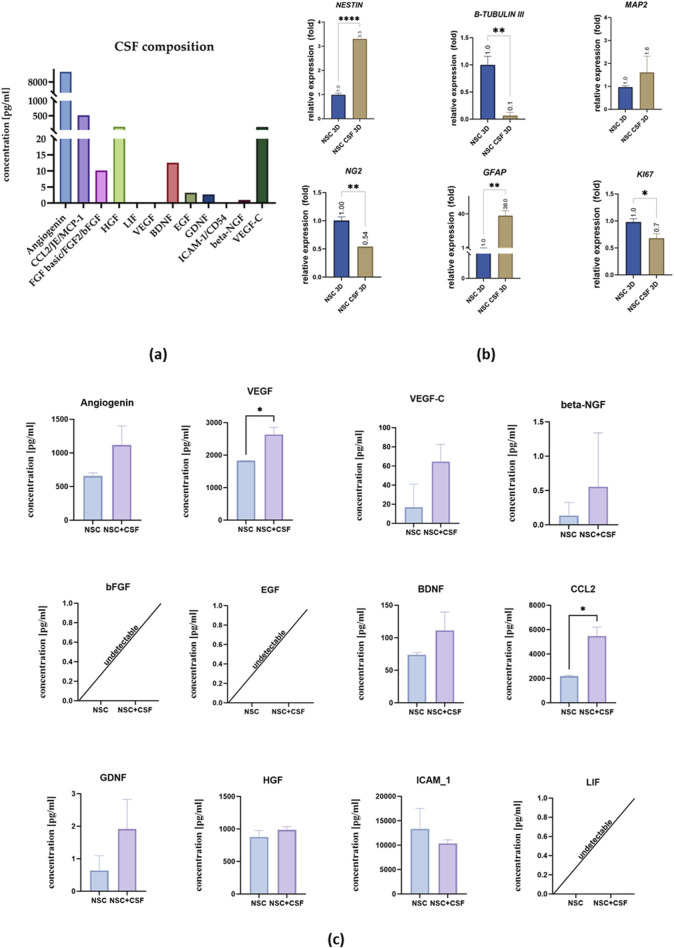
**(a)** CSF composition. **(b)** Relative expression of neural and proliferation on day 7 with RPLP0 as a reference gene and NSC cultured in a standard medium as the control group, considered as 1. **(c)** Cytokines concentration in the medium collected from control cell culture and CSF. The results of all presented experiments are expressed as mean values of three experiments ±SEM. The results are presented as mean values of three experiments ±SD, * for 0.01 < p < 0.05; for p < 0.05; ** for 0.001 < p < 0.01; *** for 0.0001 < p < 0.001; **** for p < 0.0001.

To analyze the secretory potential of NSCs, we used cells cultured in 3D conditions, which are more representative of NSCs. This eliminates the impact of analytes present in the 2D coating. We investigated two culture variants (control and CSF) and performed an analysis of neural and proliferation markers expression (Figure 6).

The concentration of selected analytes in the pooled CSF and the media collected on the last day of the experiment were analyzed (Figures 6a, c). In the pooled CSF sample, we did not observe the presence of LIF, VEGF, or ICAM-1. However, there were remarkably high concentrations of angiogenin (9,242.67 pg/mL), CCL2 (519.12 pg/mL), VEGF-C (120.2 pg/mL), and HGF (130.42 pg/mL). Additionally, we detected bFGF (10.14 pg/mL), BDNF (12.51 pg/mL), EGF (3.22 pg/mL), GDNF (2.67 pg/mL), and beta-NGF (0.94 pg/mL).

Next, we analyzed the media collected after the experiment (Figure 6c). Comparative analysis of the investigated variants showed a significantly higher concentration of VEGF (2,635 ± 218.5 pg/mL) in the CSF-treated group compared to the control (1831 ± 227.7 pg/mL), as well as CCL2 (control: 2,189 ± 76.94 pg/mL; CSF: 5,482 ± 733.2 pg/mL). There were no significant changes in the concentration of angiogenin (control: 658.5 ± 44.84 pg/mL; CSF: 1,118 ± 283.3 pg/mL), VEGF-C (control: 34.02 pg/mL; CSF: 64.62 ± 17.89 pg/mL), beta-NGF (control: 0.14 ± 0.19 pg/mL; CSF: 0.5550 ± 0.78 pg/mL), BDNF (control: 73.76 ± 3.61 pg/mL; CSF: 111.5 ± 28.12 pg/mL), GDNF (control: 0.6400 ± 0.45 pg/mL; CSF: 1.915 ± 0.91 pg/mL), ICAM-1 (control: 13,341 ± 4,205 pg/mL; CSF: 10,361 ± 722.9 pg/mL), bFGF, EGF, and LIF.

In 3D culture, on the mRNA level, we found significant differences in *NESTIN* and *β-TUBULIN III* expression between the control and CSF groups (Figure 6b). Unlike in the 2D culture, *NESTIN* expression was upregulated in the CSF group (3.3-fold), whereas *β-TUBULIN III* was downregulated (0.065 ± 0.064 fold) compared to the control. For other markers, the trends were similar to those observed in the 2D culture. Most notably, *GFAP* expression was significantly higher in the CSF group (38.03 ± 5.49 fold) than in the control. Both *NG2* and *KI67* were downregulated in the CSF group (0.54 fold and 0.68 ± 0.085 fold, respectively) compared to the control. There were no significant differences in the expression of *MAP2* between the two variants. Thus, in 3D culture, CSF treatment induces changes in gene expression among hNSCs, including upregulation of neural and astrocytic markers, and downregulation of early neuronal, oligodendrocytic, and proliferation markers compared to the control conditions.

### 3.2 Analysis of neurogenic and neuroprotective potential of hNSCs pretreated with CSF in the presence of rat organotypic hippocampal slice culture

**FIGURE 7:**
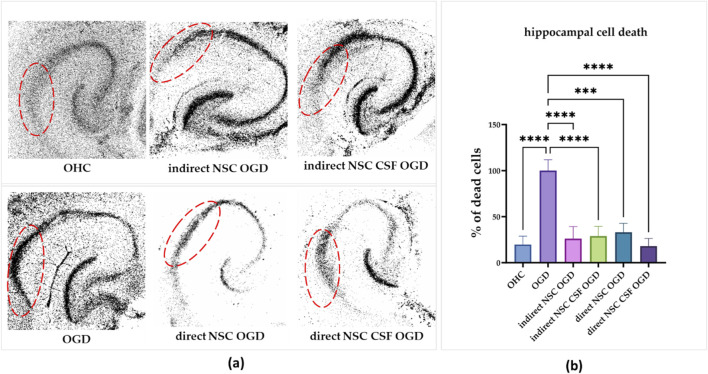
**(a)** The effect of indirect co-culture of hippocampal slices with NSC and NSC CSF on hippocampal cell death. PI incorporation into the CA1 region of the hippocampal slices (the region is marked red). **(b)** Quantitative analysis of effects of direct and indirect co-culture of hippocampal slices after OGD with NSC and NSC CSF on cell death in the CA1 zone. The results of all presented experiments are expressed as mean values ±SD. The differences were considered statistically significant when p-value <0.05. Statistical significance level **** for p < 0.0001.

The neuroprotective potential of NSCs was investigated after 24 h for both direct and indirect co-culture of control and CSF-treated cells with OGD-treated OHC (Figure 7).

Initially, we assessed the CSF and control cell survival after OGD treatment in the CA1 region of the hippocampus. The hippocampi without cell co-culture which were OGD-treated revealed the mortality of most neurons in the CA1 region after 24 h, establishing 100% cell death as the baseline (Figures 7a, b). We discovered that the co-culture with all presented variants, whether it was direct or indirect, led to a significant reduction in cell death in the CA1 hippocampal region. The greatest decrease was observed with direct co-culture (29% ± 10.4%) and indirect co-culture (18% ± 8.3%) with both CSF-treated groups. For the control group, cell death was reduced to 26% ± 13.2% with direct co-culture and 33% ± 10% with indirect co-culture. Thus, co-culture with both cell variants significantly mitigated cell death in the CA1 hippocampal region after OGD treatment, with direct and indirect co-culture showing substantial neuroprotective effects.

**FIGURE 8:**
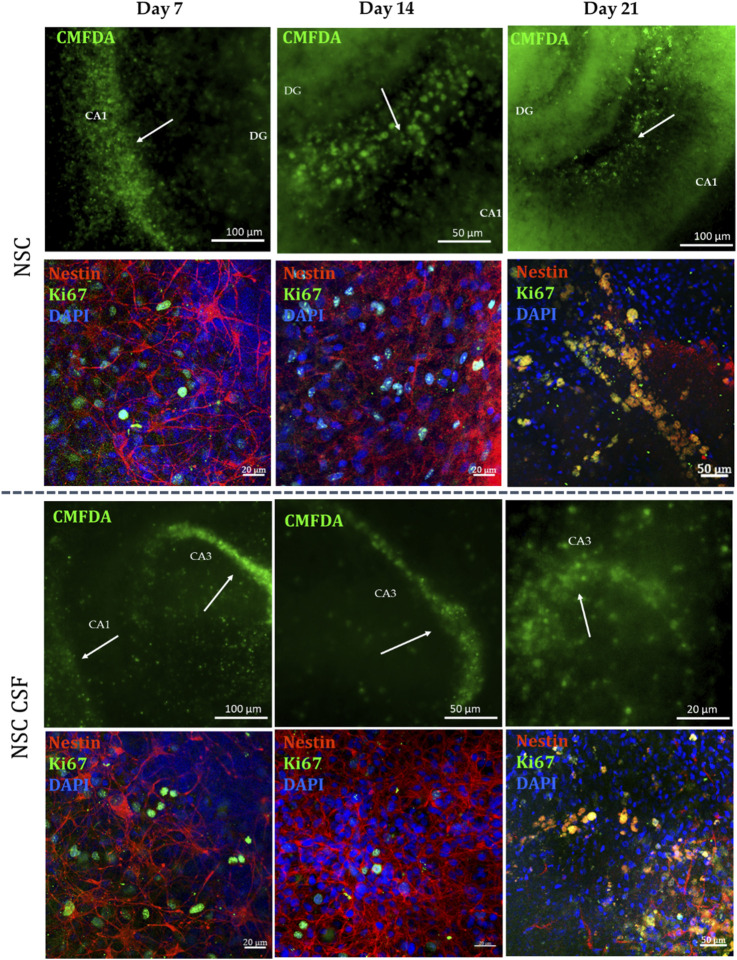
NSC localization in indirect co-culture with OGD-treated OHC after 7, 14 and 21 days of the experiment. NSC was stained with CMFDA (green) and human-reactive Nestin (red), while the proliferative cells were stained with KI67.

Next, we analyzed the localization and survival of directly transplanted cells after 7, 14, and 21 days. The first one was assessed using CMFDA-stained cells. For both CSF and control groups, we noticed the migration and incorporation of cells to the most sensitive to damaged areas of the hippocampi through all experimental time points (Figure 8). Moreover, we investigated the colocalization of selective human-reactive anti-Nestin antibody with anti-ki67 antibody. A high presence of Nestin + Ki67+ cells, as well as hippocampal Ki67+ cells was observed for both variants up to 14 days of culture. Thus, previous CSF treatment facilitated the migration and integration of transplanted cells into damaged hippocampal regions and supported early proliferation up to 14 days, as evidenced by Nestin + Ki67+ cells presence. However, there was a notable loss of Ki67 expression on day 21, indicating a decrease in hippocampal and cell proliferation in both experimental groups. Consequently, the indirect co-culture experiments were conducted for up to 14 days.

**FIGURE 9:**
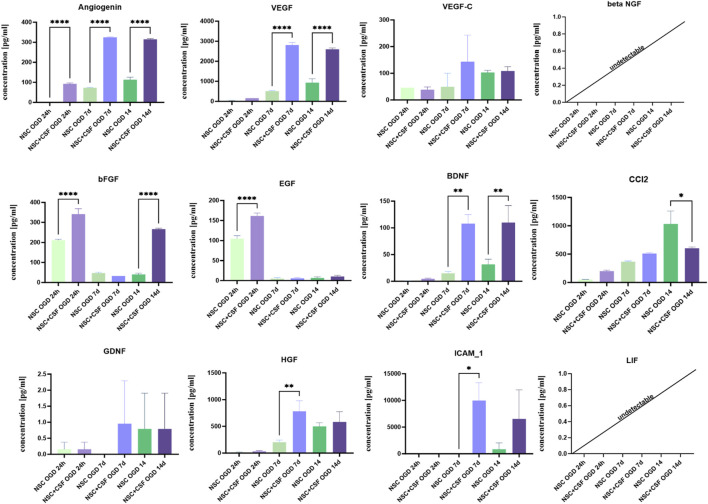
Cytokines concentration in the medium collected after co-culture of OGD-treated OHC with CSF-treated NSCs. The results are presented as mean values of three experiments ±SD. The differences were considered statistically significant when p-value <0.05. Statistical significance level * for 0.01 < p < 0.05; ** for 0.001 < p < 0.01; **** for p < 0.0001.

We investigated the concentrations of selected analytes in the media collected after 24 h, 7 days, and 14 days of indirect co-culture (Figure 9). Determining the exact secretory activity of cells cultured directly on hippocampal slices is currently beyond our technical capabilities, as the cells are incorporated into the hippocampal tissue. Therefore, the measured concentrations do not precisely reflect the cells' secretion.

Overall, for all analytes, the concentration was significantly higher in the CSF-treated groups compared to the control (Figure 9). After 24 h, the concentrations of bFGF and EGF were elevated, with significantly higher levels in the CSF group (bFGF: 341.4 ± 27 pg/mL; EGF: 161.2 ± 7.3 pg/mL) compared to the control (bFGF: 211.3 ± 4.5 pg/mL; EGF: 104.7 ± 7.5 pg/mL). A significant increase in angiogenin concentration was also observed in the CSF group (92.1 ± 4.8 pg/mL). This increase was even more pronounced after 7 days, with angiogenin levels rising to 324.2 ± 3.408 pg/mL in the CSF group compared to 72.58 ± 2.178 pg/mL in the control. While bFGF and EGF concentrations decreased after a week, we noticed significantly elevated levels of VEGF (control: 510.5 ± 15.85 pg/mL; CSF: 2,803 ± 140.1 pg/mL), BDNF (control: 14.98 ± 3.3 pg/mL; CSF: 107.8 ± 17.2 pg/mL), HGF (control: 202.5 ± 41.7 pg/mL; CSF: 780.2 ± 197.4 pg/mL), and ICAM-1 (control: undetectable; CSF: 9,987 ± 3,314 pg/mL). This trend continued after 14 days for angiogenin (control: 113.4 ± 12.4 pg/mL; CSF: 315 ± 3.4 pg/mL), VEGF (control: 936.2 ± 191.8 pg/mL; CSF: 2,597 ± 65.2 pg/mL), and BDNF (control: 31.69 ± 9.8 pg/mL; CSF: 110.0 ± 31.8 pg/mL). Additionally, CCL2 concentration was markedly higher in the control group (control: 1,031 ± 228.1 pg/mL; CSF: 602.3 ± 23.5 pg/mL), while bFGF was raised in the CSF group (control: 40.02 ± 6 pg/mL; CSF: 266.8 ± 4.7 pg/mL). The concentrations of both LIF and beta-NGF were undetectable. Thus, it seems that CSF can significantly increase the concentrations of various analytes at different time points, highlighting its regulatory effect on the secretion of neurotrophic and inflammatory cytokines.

**FIGURE 10:**
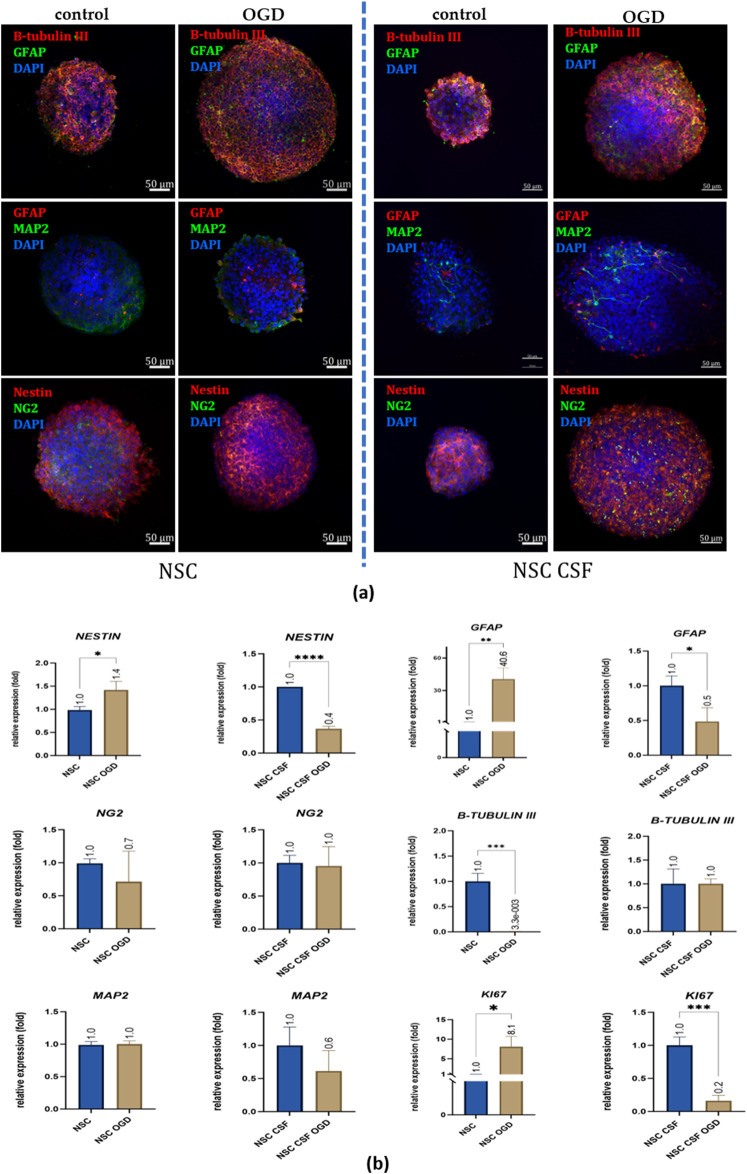
The effect of CSF and co-culture with OGD-treated OHC on NSC differentiation. **(a)** Representative images of IF staining analysis of neural markers presence. Split-channel images are available in the Supplementary Files. **(b)** Relative expression of neural and proliferation with RPLP0 as a reference gene and NSC cultured in a standard medium as the control group. The results are presented as mean values of three experiments ±SD, * for 0.01 < p < 0.05; for p < 0.05; ** for 0.001 < p < 0.01; *** for 0.0001 < p < 0.001; **** for p < 0.0001.

At the mRNA level, we found contrary results for *NESTIN, GFAP*, and *KI67* expression between control and CSF-treated cells co-cultured with OGD-treated OHC (Figure 10b). *NESTIN* expression was upregulated in the NSC OGD group (1.42 ± 0.2 fold), but downregulated in the NSC CSF OGD culture (0.37 ± 0.04 fold). However, *β-TUBULIN III* was downregulated in NSC OGD (0.003 ± 0.006 fold), with no significant changes observed in the CSF-treated culture. Interestingly, *GFAP* expression was 41-fold higher in NSC co-cultured with OGD (40.55 ± 10.34 fold) with no remarkable change seen in CSF-treated NSCs. *KI67* expression was significantly upregulated in the NSC OGD group (8.08 ± 2.6 fold) while it was downregulated in the NSC CSF OGD group (0.16 ± 0.09 fold) compared to the control. There were no significant differences in *MAP2* expression between the variants. These observations were confirmed by immunofluorescence analysis of the same markers (Figure 10a). The results suggest that CSF may modulate certain gene expression related to neural differentiation and proliferation in NSCs, changing the final effect specific to overall experimental conditions.

### 3.3 Analysis of survival, proliferation, and anti-inflammatory potential of hNSCs pretreated with CSF after transplantation into focally injured rat brain

The *in vivo* step of this study was performed to assess the survival, migration, and differentiation of the hNSCs following the intracerebral injection after ouabain-induced stroke in rats. The ouabain injury model mimics the focal brain injury. Ouabain as an inhibitor of Na/K-ATPase, causes cellular energy deprivation upon intracerebral delivery, mimicking a stroke-like cascade of events [31-32] and neuroinflammation. After the cell injection, none of the examined rats displayed any adverse symptoms, such as behavioral changes or signs of distress, indicating that the transplantation procedure was well tolerated and did not induce acute negative effects.

**FIGURE 11:**
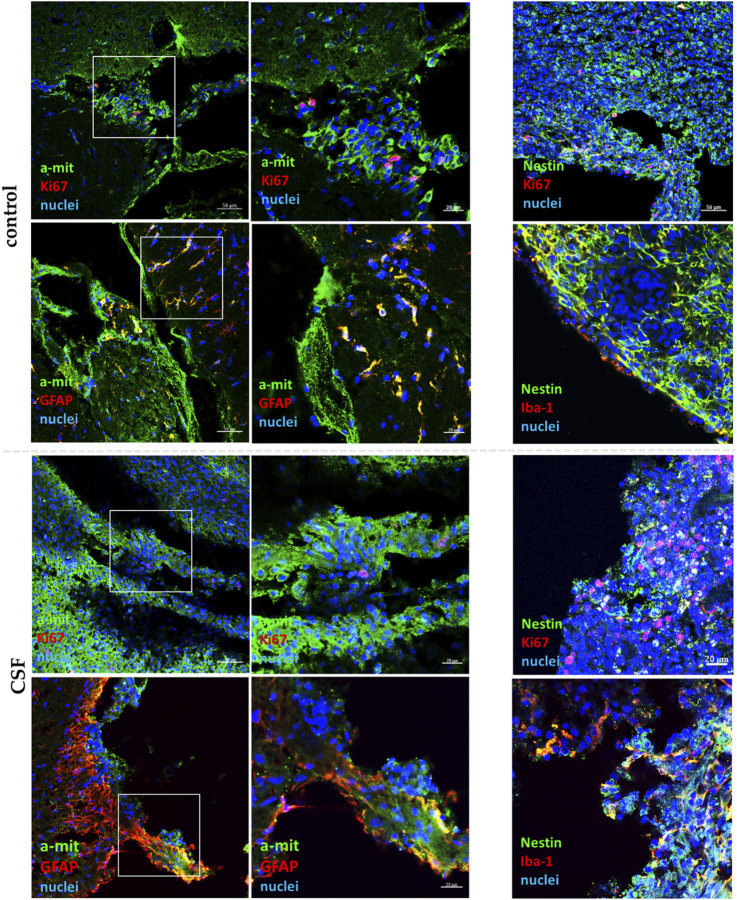
Confocal representative images of hNSC grafts *in vivo* (n = 6) in the damaged area after 7 days from transplantation. The insets present a magnification of a-mit-labeled hNSCs (green). HNSCs are stained with a-mit or Nestin (green), while the cell nuclei are labeled with Hoechst (blue).

To track and identify the transplanted cells, we used immunofluorescence analysis with antibodies specifically targeting human mitochondria (a-mit) and neural stem cell marker (Nestin). This enabled us to distinguish the human cells from the rat cells and monitor their behavior post-transplantation. The graft core was identified at the delivery site, in the stroke area (Figure 11). This suggests that the cells remained localized to the targeted area.

After 7 days, the survival of transplanted cells was significantly reduced compared to the initial number of injected cells. This reduction is likely due to a stimulation of inflammatory response. Treatment with CSF did not improve cell survival; qualitatively, it even appeared to exacerbate this, potentially due to the human origin of the CSF. We observed a low presence of a-mit + KI67+ cells, indicating limited proliferation of the transplanted hNSCs in both the control and CSF-treated groups suggesting unfavorable conditions for these cells. Most of the a-mit + cells were also positive for GFAP, a marker for astrocytes, indicating that the transplanted hNSCs were differentiating predominantly into astrocytes. Despite the significantly improved differentiation of cells treated with CSF *in vitro*, the pre-treated hNSCs did not exhibit such differentiation potential *in vivo*, which was also observed *ex vivo*. Additionally, we noted a high expression of Iba-1+ cells, which indicates activated microglia. The presence of activated microglia suggests an ongoing immune response, likely due to the injury and the presence of human-origin cells.

Overall, these findings highlight several challenges in the survival, proliferation, and differentiation of transplanted cells, especially in the context of using CSF treatment. This underscores the complexity of translating *in vitro* outcomes to *in vivo* applications, particularly in the presence of inflammatory responses and other unfavorable conditions.

## 4 Discussion

Every year, hundreds of reports emerge detailing newly discovered properties and potential mechanisms of action of NSCs in various diseases, expanding the scope of using them in clinical applications. Recognizing the pivotal role of providing NSCs with proper nutrients and the neural niche-NSC interplay, this article shifts the focus to a step into the future perspective, i.e., what happens to intrathecally transplanted NSCs, when they come into contact with the CSF.

### 4.1 Analysis of neurogenic and secretory potential of hNSCs in response to the presence of CSF

Through recent years, it has been established that CSF composition and function undergo dynamic changes throughout development, reflecting the current needs of the CNS (Gato et al., 2014; Bueno and Garcia-Fernàndez, 2016). This plays a vital role both in neurogenesis and neuropathologies (Radoszkiewicz et al., 2023a). On the other hand, the effect of this fundamental element of the brain niche on transplanted NSCs is still relatively unexplored, especially when it comes to the studies on human CSF and hNSCs (Radoszkiewicz et al., 2023a). Due to our knowledge, there is no data regarding using such concentrations of human CSF on human NSCs. Thus, we took a deeper examination *in vitro.* Our findings underscore the impact of hCSF on hNSC differentiation. Previously, Ma and coworkers presented that in NSCs originated from rat fetuses, exposition to human adult CSF resulted in glial differentiation (Ma et al., 2013). We confirmed this observation on hNSCs for both 2D and 3D cultures, where the expression of GFAP was highly upregulated. Similar insights were noted in the studies on CSF from diseased patients. For example, Buddensiek and his group reported that the human CSF obtained from patients with idiopathic normal pressure hydrocephalus promoted astrogliogenesis of hNSCs (Buddensiek et al., 2010). Similarly to our results, the group noted low differentiation abilities into mature neuronal lineage based on MAP2 expression. Nevertheless, in 2D culture, we observed a significant decrease in the expression of the early neural marker *NESTIN* upon exposure to CSF, accompanied by a remarkable increase in the early neuronal marker *β-TUBULIN III*. This effect, along with the reduced presence of NESTIN+ and SOX2+ cells, suggests a potential shift towards differentiation into neuronal lineage under CSF influence. Such dependency was noted by Henzi’s group on rat NSCs and rat CSF (Henzi et al., 2018). However, for 3D culture the expression of *NESTIN* raised after CSF treatment, while *β-TUBULIN III* was downregulated. As the expression of *NESTIN* is known to be greater in neurospheres than in the monolayer while β-TUBULIN III + cells are more frequent in 2D culture (Zheng et al., 2006), it suggests that CSF can remarkedly boost this effect and presumably strengthen the discrepancies between these two culture methods. The augmentation of *NESTIN* expression in neurospheres suggest the enhancement of NSC self-renewal and proliferative capacity in response to CSF signals (Bernal and Arranz, 2018). Indeed, we found a lower reduction of *KI67* expression in 3D culture than in the monolayer. To sum up, the differentiation effect of CSF on NSC behavior seem to be dependent not only of CSF itself, but also of cell culture methods, cell developmental origin, and microenvironmental conditions.

Moreover, CSF seems to have a great impact on NSC migratory capacity. We observed that while initial migration kinetics in scratch assay revealed enhanced scratch coverage in CSF-treated groups, this effect diminished over time and was weaker for lower CSF concentration, suggesting a dynamic modulation of NSC motion by CSF gradients. The analysis of neurite outgrowth in the scratch area further underscores this observation. Such effect has been reported previously on murine mesenchymal stromal/stem cells derived from bone marrow and rat CSF, even with using only 10% of CSF (Shokohi et al., 2017). It has also been shown that human CSF can influence fetal neural progenitor cell migration, supported by higher C-X-C chemokine receptor type 4 (CXCR4) expression (Zhu et al., 2015). The authors suggested this effect can be caused by insulin-like growth factor-1 (IGF-1), found in human CSF. Therefore, preincubation of hNSCs with CSF may augment their ability to migrate to pathological areas and increase their therapeutic potential, which is currently one of the limitations of neurological stem cell therapy.

Our investigation into the secretory potential of NSCs revealed a specific secretory profile of selected analytes in response to CSF exposure. While the pooled CSF exhibited high concentrations of both angiogenic and neurotrophic factors, CSF-treated NSCs displayed remarkably elevated secretion of VEGF and CCL2 compared to the control. This potentially suggests a role of CSF in modulating NSC secretome, which could be also correlated with migration capacity. It has been reported that the interaction between CCL2 and its receptor, CCR2, mediates the intravascular recruitment of NSC delivered through the vascular route to the ischemic brain, directing the migration of transplanted cells to the site of damage (Andres et al., 2011).

### 4.2 Analysis of hNSCs pretreated with CSF properties under *ex vivo* and *in vivo* conditions

The *in vitro* findings suggesting different therapeutic profiles of CSF-treated NSCs inspired us to check their *ex vivo* and *in vivo abilities.* To date, this is the first study that provides such insights using these two models.

The observed reduction in cell death in the CA1 hippocampal region following direct and indirect co-culture with both control and CSF-treated NSCs confirms the neuroprotective properties of these cells. Notably, the greatest reduction in cell death was observed for direct co-culture with CSF-treated NSCs, suggesting a potentiation of neuroprotective effects by CSF treatment. This exhibits the importance of the CSF in modulating NSC-mediated neuroprotection, potentially through the secretion of trophic factors and cytokines.

Furthermore, the localization and survival analysis of directly transplanted NSCs revealed their migration and incorporation into the damaged areas of the hippocampus, indicating their regenerative potential. The sustained presence of Nestin + Ki67+ cells up to 14 days post-transplantation suggests ongoing NSC proliferation within the hippocampal niche. However, the observed loss of Ki67 expression and NSC death by day 21 should be further investigated-it can be caused by the transient nature of NSC-mediated neuroprotection or a response to the toxic environment of the dead nerve tissue that culture was prolonged too extensively.

The results obtained *ex vivo* more than those obtained in the *in vitro* model suggest that preincubation with CSF significantly increases the secretory activity of NSCs upon contact with the damaged neural tissue. The findings revealed that CSF significantly modulates the concentrations of various neurotrophic and inflammatory cytokines in neural progenitor cells and NSCs. This regulatory effect is evident across different time points and highlights its potential role in enhancing neuroprotective and regenerative processes following neural injury. The reduction in the concentration of several analytes after 7 days compared to 24 h suggests a dynamic modulation of NSC secretory activity over time. It was noticed that growth factors, bFGF and EGF, were secreted immediately after damage, while most of the investigated cytokines began to be released a week after damage. Both bFGF and EGF are crucial for cell proliferation and survival, and their elevated levels suggest that CSF may enhance the initial regenerative response in neural progenitors and NSCs. This is consistent with previous studies demonstrating the roles of these factors in promoting neurogenesis and tissue repair (Radoszkiewicz et al., 2023b). The observed in our study significant increase in angiogenin and BDNF concentration after 7 days highlights the role in sustaining long-term neurogenic and angiogenic processes. Angiogenin has been shown to activate neurogenesis in the subventricular zone and support neuronal survival, aligning with the elevated levels detected in CSF-treated cultures (Subramanian et al., 2008; Steidinger et al., 2011) while BDNF was reported to produce a neuroprotective effect, promoting neuronal survival, synaptic plasticity, and neurogenesis, crucial in post-injury phase (Schäbitz et al., 2004; Chen et al., 2013). Interestingly, CCL2 levels were higher in the control group compared to the CSF-treated group. Previous studies on the impact of CCL2 on the proliferative potential and neurogenesis of endogenous NSC in a mouse model of Niemann-Pick type C disease showed a significant increase in the ability for self-renewal, proliferation, and differentiation of neurons. Moreover, it was observed that the injection of CCL2 into the mouse brain resulted in a reduction of the inflammatory state in the nervous system (Hong et al., 2015). The delayed but sustained increase in HGF in CSF-treated cultures suggests a prolonged neuroprotective response. HGF is known for its anti-inflammatory and tissue-repairing properties, and its elevated levels align with our observed benefits of CSF treatment in promoting long-term recovery (Doeppner et al., 2011; Zhang et al., 2021). These results indicate that the contact of NSC with CSF may increase the chances of survival of endogenous NSCs and lead to a more effective regeneration of the nervous system. This confirms that preincubation of NSCs with CSF stimulates their neuroprotective nature.

Our study results also shed light on how CSF regulates gene expression in NSCs co-cultured with OGD-treated OHC. It revealed significant differences between the control and CSF-treated cells, particularly for markers such as *NESTIN, β-TUBULIN III, GFAP*, and *KI67*. The upregulation of *NESTIN* and downregulation of *β-TUBULIN III* in the CSF group compared to the control after indirect co-culture with OGD-treated OHC further support the notion of differential NSC response to the CSF. *NESTIN*, a marker for neural stem cells is typically associated with neural stem cell proliferation and undifferentiated state maintenance (Sakurada et al., 2008; Park et al., 2010; Brboric et al., 2019). Our findings indicate that *NESTIN* expression was upregulated in the NSC OGD group but downregulated in the CSF-treated NSC OGD group which suggests that while OGD conditions alone may promote a proliferative and undifferentiated state, the CSF could shift the balance towards differentiation and inhibit proliferation. This is followed by the downregulation of KI67 expression of CSF-treated NSCs co-cultured with OGD, indicating a CSF modulation on NSC proliferation. Moreover, *β-TUBULIN III*, a marker of early neuronal differentiation, was significantly downregulated in the NSC OGD group suggesting a suppression of neuronal differentiation under OGD conditions with no significant changes in its expression in the CSF-treated culture. However, the notable upregulation of GFAP expression in the NSC OGD group suggests potential astrocytic differentiation under CSF influence. Interestingly, CSF treatment did not result in a significant change in GFAP expression, indicating that CSF might help preventing excessive astrocytic proliferation and potentially detrimental gliosis. Thus, CSF can significantly alter the gene expression profile of NSCs under OGD conditions, promoting a possibly more favorable environment for neural repair and regeneration.

Furthermore, the *in vivo* experiments preliminarily demonstrated the efficacy of hNSC transplantation in a rat model of ouabain-induced stroke. The absence of adverse symptoms following cell injection, coupled with the successful identification of transplanted cells within the stroke area after 7 days, highlights the feasibility of utilizing hNSCs as a therapeutic intervention for stroke-induced brain injury in the future. Moreover, the ability of transplanted hNSCs to migrate to the targeted region underscores their potential for neural tissue repair and functional recovery. However, the *in vivo* environment also presented significant hurdles. One of the most notable observations was the markedly reduced survival rate of the transplanted cells, likely due to an inflammatory response triggered by the transplantation of human-origin cells to the rat brain. The inflammation created a hostile environment, leading to increased cell death. Indeed, the transplanted human cells were shown to have poor long-term survival in a xenobiotic environment, such as rat brains (Hovakimyan et al., 2012; Tierney et al., 2020). Additionally, both control cells and hNSCs pre-treated with CSF showed low proliferation. Moreover, while CSF treatment significantly enhanced cell differentiation *in vitro*, the pre-treated cells did not exhibit the same differentiation potential *in vivo*. However, the majority of transplanted, non-treated a-mit + hNSCs were also GFAP positive, indicating differentiation into astrocytes, which is consent with previous studies (Rota Nodari et al., 2010; Lee et al., 2015; De Gioia et al., 2020). Astrocytes play several critical roles in the brain, including supporting neuronal function, maintaining the blood-brain barrier, and modulating synaptic activity (Siracusa et al., 2019; Gradisnik and Velnar, 2023). Thus, these cells might contribute to creating a supportive environment for neural repair and regeneration. However, they also elicit a microglial response, which needs to be carefully investigated. Future studies should focus on long-term tracking of these cells to better understand their potential for integration, proliferation, and differentiation of transplanted cells over time.

Taken together, this study highlights the potential of CSF to modulate NSC behavior, contributing to our understanding of brain function and disease mechanisms. *In vitro*, CSF treatment significantly influenced NSC proliferation, migration, and differentiation, with significant changes in protein and mRNA expression levels. *Ex vivo*, the analysis demonstrated that CSF maintained the neuroprotective potential of NSCs, influenced their proliferation and differentiation, and remarkably enhanced the secretion of various neurotrophic and inflammatory cytokines. *In vivo*, in a rat experimental stroke model, the transplanted NSCs showed targeted migration to the stroke-affected area without causing adverse effects. However, the study showed the limited survival and proliferation of these cells as well as the presence of activated microglia indicating an ongoing immune response. Further studies should focus on the extended survival, differentiation, and integration of these cells in the brain, as well as their impact on functional recovery and the host immune response over longer periods. This study lays the groundwork for developing new treatments for neurological disorders, potentially leveraging the unique properties of CSF to enhance NSC efficacy.

## 5 Conclusion

➢ CSF seems to have a vital role in inhibiting the proliferation and stemness properties while stimulating further differentiation of NSCs;
➢ CSF appears to change the secretory potential of NSCs, especially after contact with the damaged neural tissue;
➢ CSF contains factors that seem to modulate NSC behavior and support regeneration or even restoration of the damaged neural tissue;
➢ Following the ischemic injury, NSCs secrete factors that induce cell proliferation; after a longer period, they start releasing immunomodulatory, proangiogenic, and neuroprotective factors, which confirms our previous observations;
➢ NSCs exposed to human CSF presence exhibit decreased survival/proliferation and increased immunogenicity after intraparenchymal transplantation into the rat brain (xenograft) which is connected to additive immunogenic CSF impact;
➢ It is crucial to further study the interactions between NSCs and CSF and optimize the conditions in which CSF could support NSCs, promote regeneration and restoration, and improve targeted cellular therapy in CNS disorders.

## Data Availability

The original contributions presented in the study are included in the article/Supplementary Material, further inquiries can be directed to the corresponding author.

## Abbreviations

hNSCs, human neural stem/progenitor cells; CSF, cerebrospinal fluid; OGD, oxygen-glucose deprivation; OHC, organotypic hippocampal slices culture; PI, propidium iodide; CNS, central nervous system; DMSO, dimethyl sulfoxide; CXCR4 C-X-C Motif Chemokine Receptor 4; ESCs, embryonic stem cells; NGF, nerve growth factor; PFA, paraformaldehyde; LIF, leukemia inhibitory factor; BDNF, brain-derived neurotrophic factor; HBSS, Hanks’ Balanced Salt Solution; DMSO, dimethyl sulfoxide; bFGF, basic fibroblast growth factor; EGF, epidermal growth factor; VEGF, vascular endothelial growth factor; IGF, insulin-like growth factor; MAP2, Microtubule-associated protein 2; GFAP, Glial fibrillary acidic protein; KI67, marker of proliferation Ki67; HGF, hepatocyte growth factor.
