# Supplementary figures and images for: Unraveling the impact of human cerebrospinal fluid on human neural stem cell fate
Source: Front Cell Dev Biol. 2025 Mar 13;13:1527557. doi: 10.3389/fcell.2025.1527557 (PMC11950821; doi:10.3389/fcell.2025.1527557)

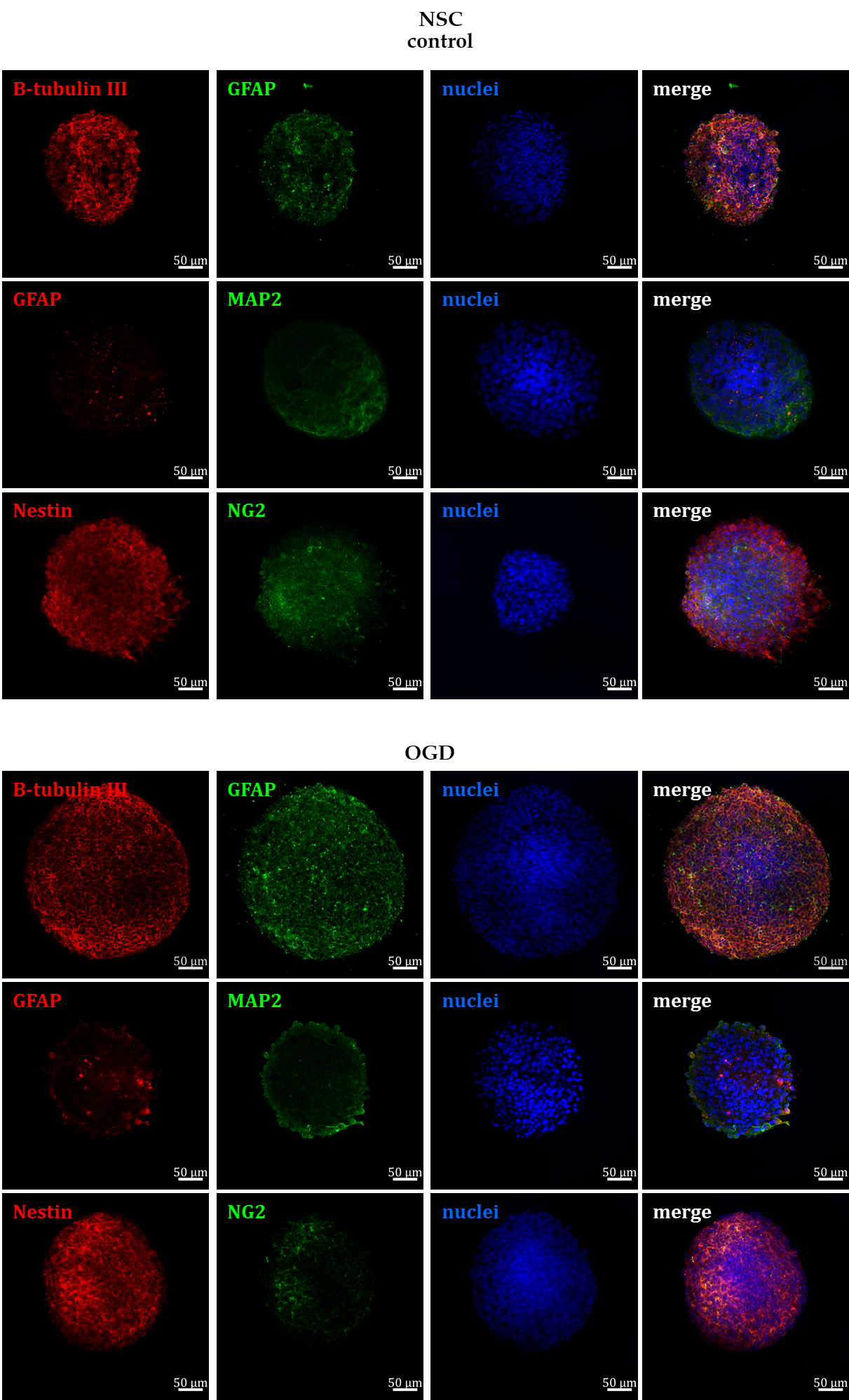

Supplement: Supplementary file 1 [file Image3.tif]

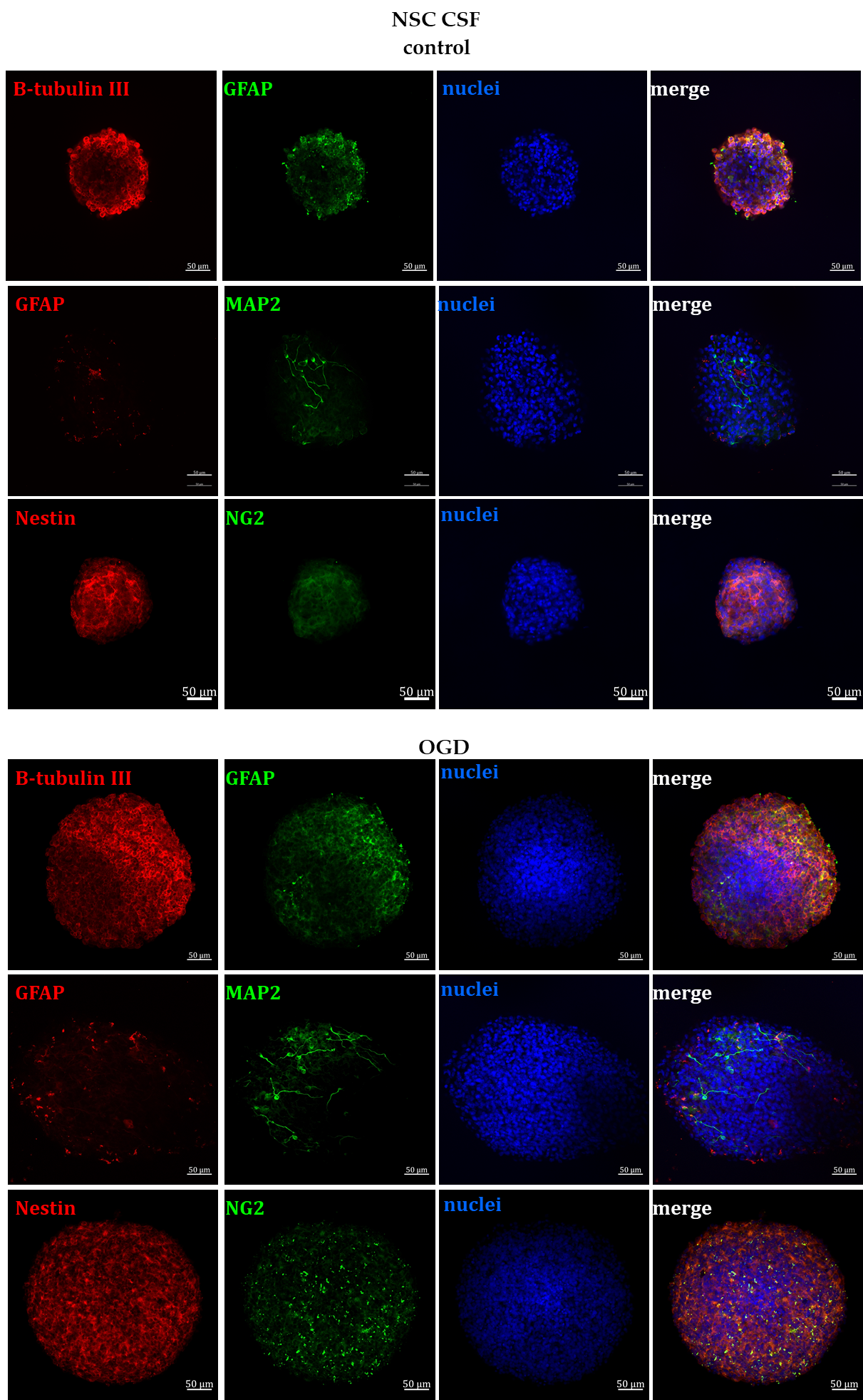

Supplement: Supplementary file 2 [file Image4.tif]

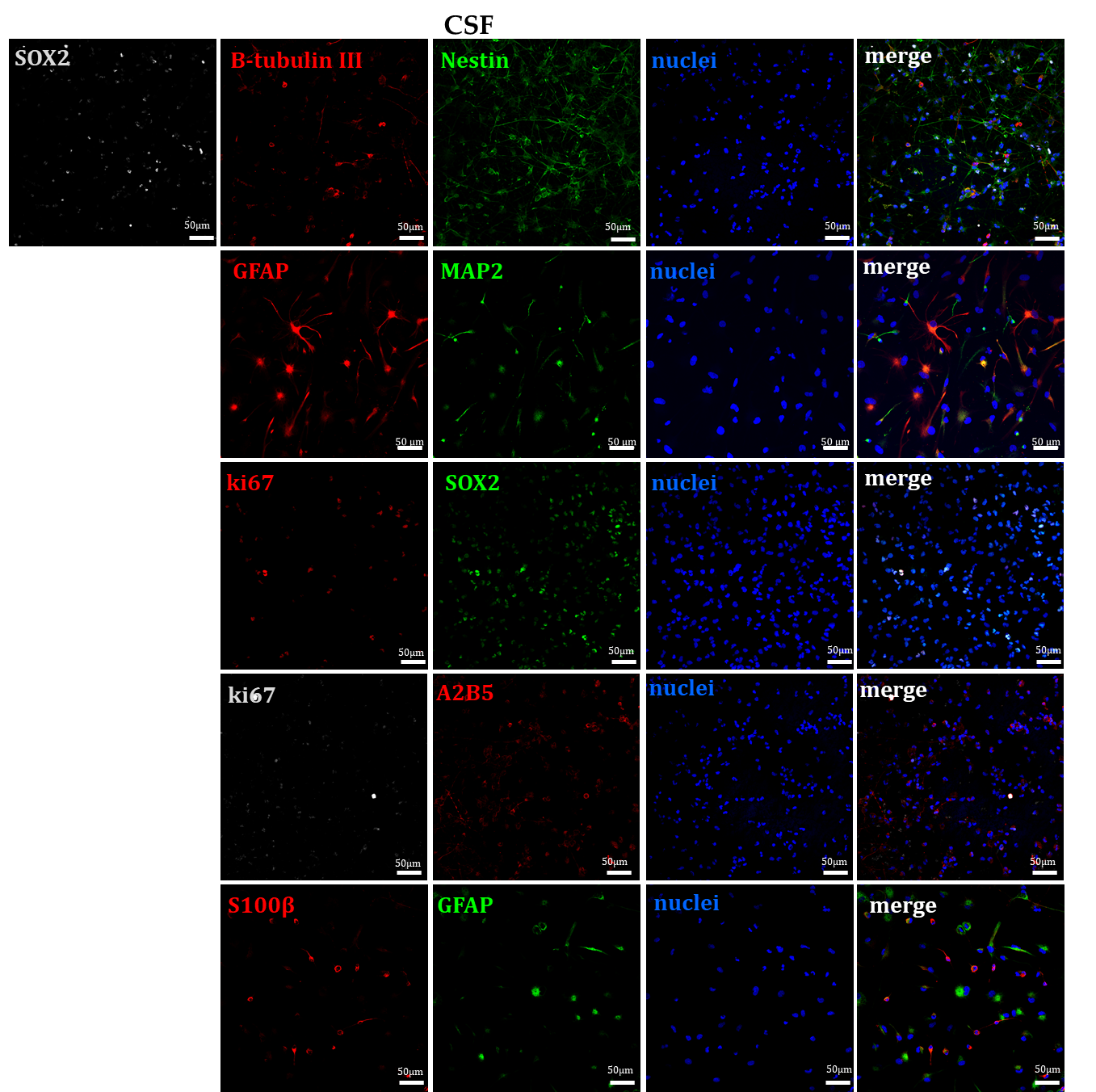

Supplement: Supplementary file 3 [file Image2.tif]

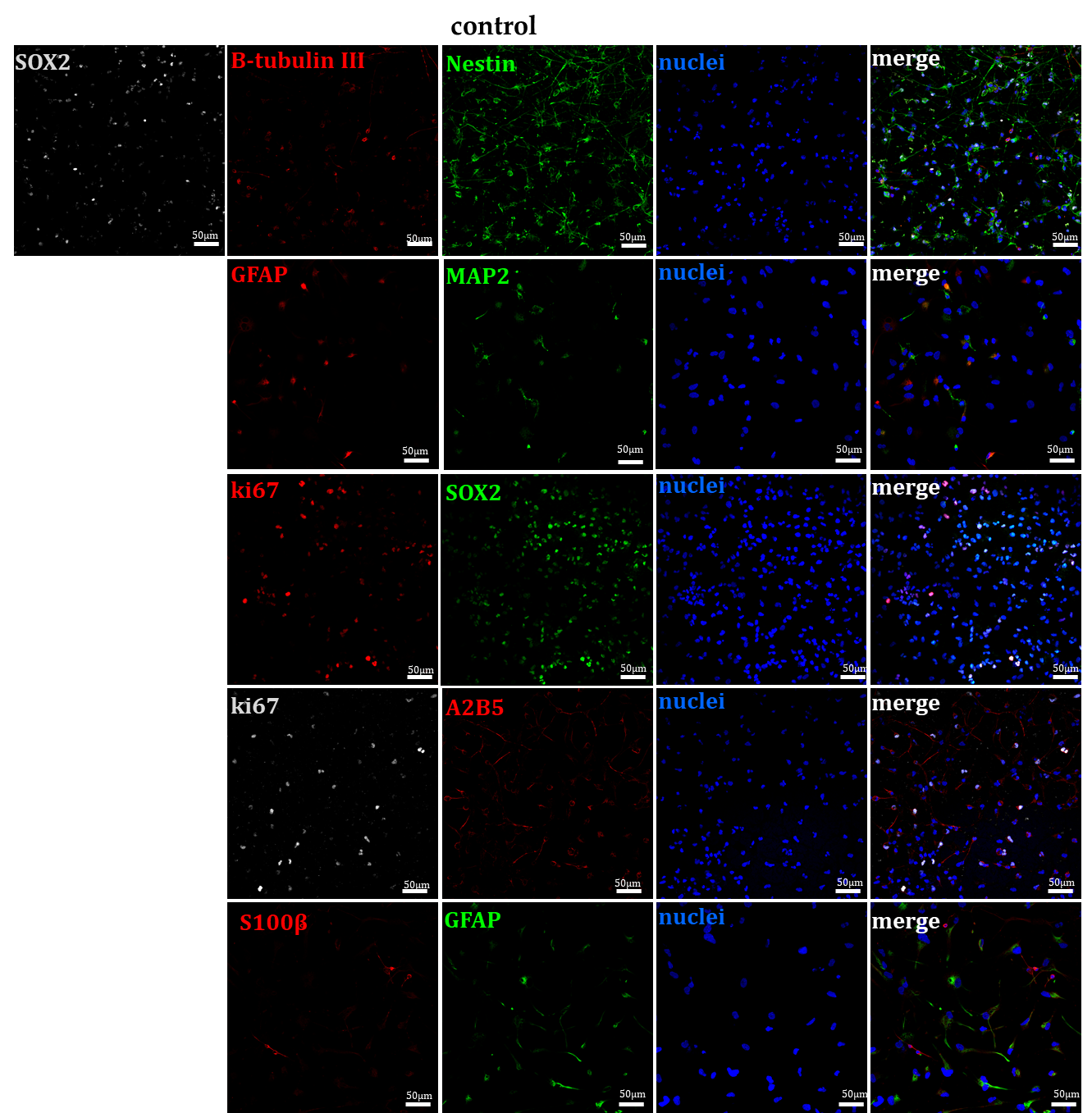

Supplement: Supplementary file 4 [file Image1.tif]
